# A Macrocyclic Peptide that Serves as a Cocrystallization Ligand and Inhibits the Function of a MATE Family Transporter

**DOI:** 10.3390/molecules180910514

**Published:** 2013-08-30

**Authors:** Christopher J. Hipolito, Yoshiki Tanaka, Takayuki Katoh, Osamu Nureki, Hiroaki Suga

**Affiliations:** 1 Department of Chemistry, Graduate School of Science, the University of Tokyo, 7-3-1 Bunkyo-ku, Tokyo 113-0033, Japan; E-Mails: hipolito@chem.s.u-tokyo.ac.jp (C.J.H.); katoh@chem.s.u-tokyo.ac.jp (T.K.); 2 Department of Biophysics and Biochemistry, Graduate School of Science, the University of Tokyo, 2-11-16 Yayoi, Bunkyo-ku, Tokyo 113-0032, Japan

**Keywords:** *in vitro* selection, cocrystallization ligand, random non-standard peptide integrated discovery (RaPID), macrocyclic peptide, PfMATE

## Abstract

The random non-standard peptide integrated discovery (RaPID) system has proven to be a powerful approach to discover *de novo* natural product-like macrocyclic peptides that inhibit protein functions. We have recently reported three macrocyclic peptides that bind to *Pyrococcus furiosus* multidrug and toxic compound extrusion (PfMATE) transporter and inhibit the transport function. Moreover, these macrocyclic peptides were successfully employed as cocrystallization ligands of selenomethionine-labeled PfMATE. In this report, we disclose the details of the RaPID selection strategy that led to the identification of these three macrocyclic peptides as well as a fourth macrocyclic peptide, MaD8, which is exclusively discussed in this article. MaD8 was found to bind within the cleft of PfMATE’s extracellular side and blocked the path of organic small molecules being extruded. The results of an ethidium bromide efflux assay confirmed the efflux inhibitory activity of MaD8, whose behavior was similar to that of previously reported MaD5.

## 1. Introduction

Transmembrane proteins are comprised of hydrophobic regions that span the membrane lipid bilayer and hydrophilic regions that are exposed on one of two sides of the membrane. Hydrophilic surfaces are critical for specific protein-to-protein contact in a crystal lattice; therefore, high-quality crystals of transmembrane proteins, with their reduced hydrophilic surfaces, are difficult to obtain. The hydrophobic surfaces promote aggregation and precipitation. This propensity of transmembrane proteins to aggregate and precipitate extends to intracellular proteins like HIV-1 Rev, which spontaneously polymerizes into filaments, and its successful crystallization required the creation and use of a cocrystallization ligand [1]. In addition, transmembrane proteins involved in the translocation of molecules across the membrane could be highly conformationally dynamic, which is another characteristic that is detrimental to crystallization.

Detergents and other additives are often used to help solubilize the target protein and promote crystallization by modifying the protein surface to increase the potential for specific intermolecular contact [2]. Larger engineered additives (for example, *in vitro* selected binding proteins) can immobilize dynamic regions to increase the homogeneity of the protein sample [3] and mediate lattice formation to prevent problems like twinning defects [4]. Such proteinaceous cocrystallization ligands have been referred to in the literature as co-crystallization proteins (CCPs) [5]. Fragments of natural binding partners, *in vitro* selected antibody Fv (fragment variable) fragments [6], antibody Fab (fragment antibody binding) fragments [7], and DARPins (designed ankyrin repeat proteins) [8,9] have been successfully employed as cocrystallization ligands.

The display techniques employed in the *in vitro* selection for Fab fragments and DARPins, phage display [10] and ribosome display [11], respectively, have been used to successfully identify cocrystallization ligands [8,9], but possess certain limitations. Libraries constructed using phage display are often limited by the infection efficiency to suitable bacterial hosts; in general, 10^10^ or less unique molecules would represent a practical library size [12]. Unlike phage display, ribosome display does not use host cells, *i.e.*, completely *in vitro*, and allows researchers to perform selections using library sizes on the order of 10^13^ unique molecules. However, ribosome display is dependent on the stability of the mRNA-ribosome-peptide ternary complex; therefore, applicable selection conditions are restricted to magnesium-containing buffers, *i.e.*, magnesium-free buffers or buffers containing other metal ions that potentially disturb the ternary complex cannot be used. Moreover, successful demonstrations of high affinity ligands using ribosome display have, thus far, been limited to the use of libraries based on a relatively large protein scaffold such as DARPin, and therefore, this simply represents an alternative to antibodies or Fab fragments.

Macrocyclic peptides are a class of binding molecules that could potentially assist with the crystallization of transmembrane protein by stabilizing a specific protein confirmation. The selection for high affinity macrocyclic peptides is accomplished using an *in vitro* display platform, in this case, mRNA display [13,14]. We have recently developed this technology, referred to as the random non-standard peptide integrated discovery (RaPID) system [15], where a genetic code reprogramming method [16] based on flexizyme technology [17,18,19,20,21,22] is combined with mRNA display. The RaPID system enables us to select high affinity macrocyclic peptides from a large library consisting of more than 10^12^ members [23]. Most importantly, the selected unique macrocyclic peptides are closed by a non-reducible thioether bond, and often exhibit remarkably high affinity with single-digit or sub nM dissociation (or inhibitory) constants against a target protein chosen for selection [15,24,25]. The RaPID system is commonly used for discovering inhibitors against therapeutic targets; however, the above properties observed in the selected macrocyclic peptides suggest an application to developing cocrystallization ligands as well as inhibitors from selected macrocyclic peptides.

We have chosen *Pyrococcus furiosus* multidrug and toxic compound extrusion (PfMATE) transporter as a model protein to assess the use of macrocyclic peptides as cocrystallization ligands. PfMATE is a transmembrane protein homologous to NorM-VC, whose crystal structure has been determined at a 3.65 Å resolution [26]. More recently, structures of the MATE family transporter NorM-NG have been solved in the apo form and in complexes with three translocation substrates at 3.5–3.6 Å resolution [4]. Additionally, the authors report a Cs^+^-bound form, which supports the proposed Na^+^ anti-porter mechanism. Their structure determination suffered from twinning defects, which was resolved by cocrystallization with an engineered monobody that mediated lattice formation. Nevertheless, some drug-resistant bacterial MATE transporters and human MATE transporters, such as hMATE1 [27] and hMATE2-K [28], belong to a group of MATE family transporters that use a proton-driven anti-porter mechanism. It was determined that PfMATE also uses such a proton-driven anti-porter mechanism [29]. Protonation of D41 of PfMATE in an outward-open state causes a kinking of transmembrane helix 1 (TM1), which promotes the release of the translocation substrate and conversion back to an inward-open state. Considering the structural flexibility of TM1, an alpha-helix facing the center channel of the transporter, as well as the structural flexibility of the gross morphology of the entire protein, crystallization of PfMATE would greatly benefit from the presence of a conformation-specific cocrystallization ligand. *In vitro* selected macrocyclic peptides have the potential to play dual roles in not only cocrystallization of PfMATE as a conformation-specific cocrystallization ligand but also in inhibiting PfMATE’s drug extrusion mechanism. Thus, we have explored such a possibility in this work. 

Originating from the selection presented in this article, three macrocyclic peptides, MaL6, MaD3S, and MaD5, were previously reported in the article by Tanaka *et al*. [29]. The cocrystal structures of these three macrocyclic peptides were solved in complex with PfMATE to 3.0–2.4 Å resolutions. All three macrocyclic peptides appeared to stabilize a single conformational state thereby facilitating cocrystallization. By using an ethidium bromide accumulation assay, the macrocyclic peptides’ inhibition of pump activity was confirmed. This inhibitory activity is evidence of solution phase binding and locking of the outward-facing state of PfMATE. Here, we provide a more in-depth discussion about the strategy used for the identification of various macrocyclic peptides that specifically bind to PfMATE. Moreover, we here describe another macrocyclic peptide, MaD8, which was found in the aforementioned selection campaign and is exclusively discussed in this article.

## 2. Results and Discussion

### 2.1. RaPID Selection of PfMATE-Binding Macrocyclic Peptides

#### 2.1.1. RaPID-Displayed Cyclic Peptide Library

The RaPID selection was carried out according to the original protocol reported in the literature [25] with minor modifications (Figure S1). The initial mRNA libraries were designed with an AUG initiator codon that is reprogrammed with an initiator tRNA charged with either *N*-(2-chloroacetyl)-L-phenylalanine or *N*-(2-chloroacetyl)-D-phenylalanine by using the methionine-deficient Flexible *in vitro* Translation (FIT) system [20]. The AUG initiator codon is followed by a random region of 7–15 NNK codons (where N represents any of the four RNA bases and K represents either U or G), which codes for 7–15 random proteinogenic amino acid residues. There are three important points regarding the use of the NNK codon. First, the NNK codon cover all 20 proteinogenic amino acids including methionine coded by AUG. As a result of removing methionine from the translation mixture, we have empirically found that isoleucine is generally misincorporated at the elongation AUG positions *in lieu* of methionine (Suga laboratory, unpublished data). Second, it should also be noted that the UAG (amber) stop codon could also appear in the random region. Statistically, 38% of the library containing 15 consecutive NNK codons will be free of premature stop codons [(15/16)^n^ where n is the number of consecutive NNK codons]. Finally, a UGU coding for a cysteine can appear in the random region. The 3'-end of the mRNA random region is deliberately flanked by a UGU codon that codes for a cysteine; this residue is intended for use in the macrocyclization reaction [30]. Although this downstream Cys is incorporated to ensure the presence of a nucleophile for the macrocyclization of the peptide, a recent study by Iwasaki *et al*. revealed that macrocyclization occurs between the *N*-terminal chloroacetyl group and the nearest neighboring cysteine except for the first downstream position appearing in the random region [31]; therefore, macrocyclization would employ the *N*-terminal chloroacetyl group and a cysteine appearing in the random region preferentially over the deliberately positioned cysteine flanking the random region.

With these points in mind, the NNK codon was chosen for the random region over the NNU codons [15] and the NNC codons [24] for two reasons. First, NNK codes for all 20 amino acids, 19 of which are available for elongation. Excluding the four amino acids glutamine, glutamate, tryptophan, and especially lysine would be detrimental to the goal of obtaining charge-rich macrocyclic peptides. Second, Hayashi *et al*. used the NNK codon without compensating for the amber codon or the vacant AUG codon box and obtained active inhibitors from the (NNK)_12_ library showing that it is possible to obtain macrocyclic peptides from these libraries of longer peptide sequences without reassignment of the vacant elongation codon boxes [25]. Modification of the reprogrammed genetic code can be performed in future selections using codon reassignment of the non-coding codon boxes UAG to increase the effective macrocyclic peptide library size by removing premature stops, respectively.

To covalently link the mRNA to a nascent macrocyclic peptide, a DNA primer bearing the 3'-PEG-linked puromycin and complementary to the 3'-end of the mRNA templates was ligated to the 3'-end of mRNAs. *In vitro* translation of a macrocyclic peptide library containing 7–15 amino acids of random identities was initiated with *N*-(2-chloroacetyl)-L-phenylalanine (^L^F-library) using the puromycin-DNA-RNA conjugate. *In vitro* translation of a second macrocyclic peptide library also containing 7–15 amino acids of random identities was initiated with *N*-(2-chloroacetyl)-D-phenylalanine (^D^F-library) [24,25]. By virtue of the absence of release factor 1, the puromycin established a covalent linkage between the constant C-terminal linker sequence (a glycine-serine triple repeat) appeared after the random peptide region and the cognate mRNA sequences with an efficiency of at least 30% [25]. We estimate that, between the appearances of UAG stop codons and mRNA display efficiency, we still obtain macrocyclic peptide libraries with at least 10^12^ unique members each.

#### 2.1.2. Initial Selection with a Binding Step at 4 °C

Wildtype PfMATE was constructed with a C-terminal His_6_-tag to facilitate purification and immobilization [29]. The His_6_-tagged PfMATE protein was immobilized on Dynabeads^®^ His-Tag Isolation & Pulldown magnetic beads (Invitrogen). The RaPID selection for PfMATE-binding macrocyclic peptides was divided into two consecutive sub-selections. In both sub-selections, the ^L^F- and ^D^F-libraries were independently used in the initial round, as well as their respective progeny libraries in the subsequent rounds. The binding step of the first sub-selection was carried out at 4 °C to promote binding of macrocyclic peptides with modest to strong affinity for PfMATE. Prior to incubation with the bead-bound PfMATE, His_6_-tagged translation components and bead-binding macrocyclic peptides-mRNA conjugates were removed from the solution containing the macrocyclic peptide library by first incubating the solution with PfMATE-free magnetic beads. This process, referred to as pre-clearance, was performed three times. For the first round, the macrocyclic peptide library remaining after pre-clearance was incubated with bead-bound PfMATE for one hour at 4 °C with rotation. Subsequently, the beads were washed three times to remove weak and non-specific binders. Starting from round two, the duration of the binding step was shortened to thirty minutes. The first signs of enrichment were observed in the 5th round from the ^L^F-library (Figure S2A) and the 4th round from the ^D^F-library (Figure S2B).

**Figure 1:**
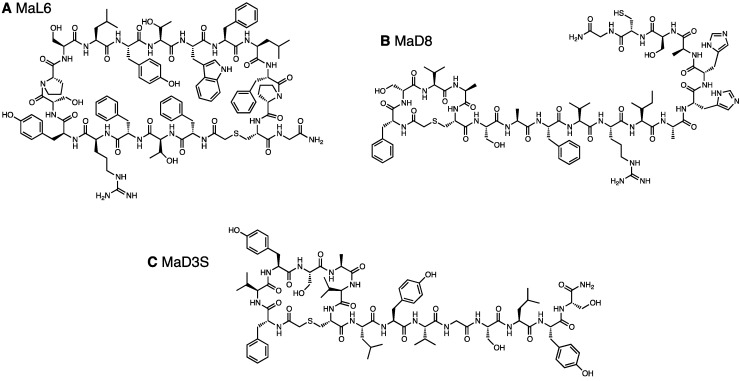
Chemical structure of *in vitro* selected macrocyclic peptides that bind to PfMATE. (**A**) MaL6, (**B**) MaD8, and (**C**) MaD3S.

Clearly elevated cDNA recoveries, indicating enrichment, were obtained in the round 6 of each library, where the observed recovery percentages were 7.5% and 4.5% of the input DNA from ^L^F-library (Figure S2A) and ^D^F-library (Figure S2B), respectively. No further enrichment was observed in 7th round for both libraries, leading to our decision to halt the selection at this round. The cDNA obtained from the pools of the 7th round (referred to as ^L^F- and ^D^F-round 7 pools) were cloned, and individual sequences were determined (Table 1). A single kind of macrocyclic peptide, referred to as MaL6 (Figure 1A), appeared in the highest frequency in the ^L^F-round 7 pool. On the other hand, no conservation of peptide sequence was found in the ^D^F-round 7 pool even though the same selective pressure was given to both libraries. This could be attributed to a possibility that the initial ^D^F-library might contain a greater variety of macrocyclic peptides exhibiting modest affinities to PfMATE.

#### 2.1.3. Increasing Stringency with a Binding Step at 37 °C

To deplete the appearance of macrocyclic peptides with modest affinity to PfMATE from the enriched libraries of the later rounds, we employed a selection for PfMATE-binding macrocyclic peptides with higher affinity than those found in the first sub-selection using the original libraries by elevating the temperature of the PfMATE binding step to 37 °C, instead of 4 °C. Enrichment was observed in both ^L^F- and ^D^F-Round 4 (Figure S2C,D, respectively; note that to distinguish currently discussed rounds from the rounds of the aforementioned selection attempt, the capitalized term “Round” is used). The recovered DNA percentages increased to 10.9% and 9.4% for both pools at Round 6 (Figure S2C,D, respectively), which are values sufficiently high enough for us to halt the selection.

**Table 1:** Compiled peptide sequences from the 7th round of sub-selection 1, Round 6 of sub-selection 2, and c-Round 6 of sub-selection 2. Clones with the MaL-prefix originated from the ^L^F-Library and clones with the MaD-prefix originated from the ^D^F-Library. Clone residues are shown from the non-standard initiator amino acid represented by AcF for *N*-chloroacetyl-L-phenylalanine and Acf for *N*-chloroacetyl-D-phenylalanine to the engineered Cys. MaL2 and MaD5 were the result of frameshift mutations and have lost their engineered Cys, (Gly-Ser)_3_ linker, and stop codon.

Anti-MATE Selection Clone Sequences					
Name	Sequence	Round 7	Round 6	c-Round 6	Frequency of appearance
MaL1	AcFSTFCYFPTELLLLAC	1/9			1/32
MaL2	AcFTDCLHARWIFPRVRQRQRQLGRGAEKSLVNSRP		1/11		1/32
MaL3	AcFVYSAVCLYVGSLYPC		2/11	4/12	6/32
MaL4	AcFTFRDVWIFYGSLLSRC		1/11		1/32
MaL5	AcFLYNAYCLWLAYCVNSC				2/32
MaL6	AcFTFRYSPSLYTWFLFPC	4/9	7/11	3/12	14/32
MaL7	AcFWTVASWGLVALDFVAC	1/9			1/32
MaL8	AcFTHPIFCYPSADLC	1/9			1/32
MaL9	AcFTDCLHARWIFPRVC	1/9			1/32
MaL10	AcFTYSAFCYAIANIAYC			3/12	3/32
MaL11	AcFAYECMWLTLPASWPPC	1/9			1/32
MaD1	AcfQWQCHIFTNLALTC		6/11	1/9	7/27
MaD2	AcfHPVNCTNLWAAIRLAC	1/7			1/27
MaD3	AcfVYSAVCLYVGSLYSC	1/7	2/11	3/9	6/27
MaD4	AcfLYNAYCLWLAYCVNSC	1/7			1/27
MaD5	AcfVYSAVCYSIAAAAAAARTGGGKITS		1/11	2/9	3/27
MaD6	AcfVDASACSFVNLWLTC	1/7		1/9	2/27
MaD7	AcfIECQTLVYLSLIPHNC	1/7			1/27
MaD8	AcfSVACSAFVRIAHHASC	1/7			1/27
MaD9	AcfTTYSAFCYAIANIAYC	1/7			1/27
MaD10	AcfTYSAFCYAIANIAYC		1/11	2/9	3/27
MaD11	AcfVNTSVCLFACWVNSC		1/11		1/27

#### 2.1.4. A Chemically Synthesized, *in Vitro* Selected Competitor

Since MaL6 was repeatedly identified from the initial selection attempt using the ^L^F-library, its binding site might represent a high affinity peptide-binding site in PfMATE. Kossiakoff and coworkers report that antigenic interaction hot spots appear in selections for synthetic antibodies, and we applied a similar remedy as the one that they presented [3]. To deplete RaPID-displayed MaL6 or other modest affinity macrocyclic peptides related to this potential hot spot from this second sub-selection, a selection round, starting from the libraries obtained after Round 5, was performed in the presence of chemically synthesized MaL6 (Figure 1A) that is not conjugated to coding mRNA and bears an additional C-terminal glycine-carboxamide. The chemically synthesized MaL6 was added to a final concentration of 32 µM, which far exceeded the concentration of RaPID-displayed macrocyclic peptides (<2 µM). The recoveries from this MaL6-competitive Round 6 (c-Round 6) pools were 6.2% and 4.8% from the respective Round 5 pools (Figure S2C,D, respectively). These percentage values obtained from the c-Round 6 were lower than the corresponding recoveries from the non-competitive Round 6, suggesting that the recovery of macrocyclic peptide-mRNA conjugates that bind to the MaL6-competitive site could be reduced by the addition of chemically synthesized MaL6.

The ^L^F- and ^D^F-Round 6 pools as well as ^L^F- and ^D^F-c-Round 6 pools were cloned, and DNA amplified from arbitrarily picked colonies were subjected to sequencing (Table 1). The sequence convergences found in the ^L^F- and ^D^F-Round 6 pools were higher than those found in the ^L^F- and ^D^F-round 7 pools selected at 4 °C. Interestingly, a single Cys residue frequently appeared in the random region of the isolated clones, indicating that the selection favored peptides with minicycle head motifs. All identified clones, having undergone a frame shift mutation or not, bore a macrocyclic backbone. The PfMATE-binding ability of the individual clones was first verified by the single-clone display (Figure S3), showing that the majority of the clones were active with respect to binding to PfMATE. From these active clones, we chose three clones, referred to as MaD8 (Figure 1B), MaD3S (a chemical derivative of MaD3 lacking the engineered Cys, Figure 1C), and MaD5, in addition to MaL6 for co-crystallizations with PfMATE. MaD8 was also synthesized with an additional C-terminal glycine-carboxamide. Since MaD3S, MaD5, and MaL6 had been described elsewhere [29], we here report characterization of MaD8.

### 2.2. Co-Crystallization of the MaD8-PfMATE Complex

**Figure 2:**
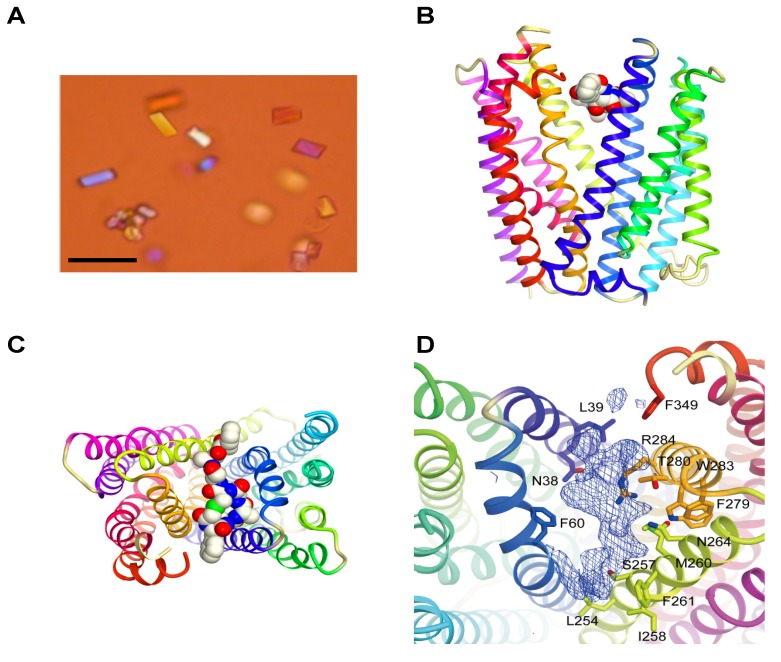
Crystals and crystal structure of the MaD8-PfMATE complex. (**A**) Crystals of the MaD8-PfMATE complex. The scale bar represents 50 µm; (**B**) Side view of the complex structure. Based on the omit map, MaD8, in ball representation, is shown bound between the N- and C-lobes of a selenomethionine-derivative of PfMATE, which is represented in cartoon format; (**C**) Top view of the complex structure. A portion of the C-terminal region of the peptide was not visible in the crystal structure; (**D**) MaD8-binding region. Omit map electron density shows possible π-stacking interaction with the sidechain of F349 of PfMATE.

The MaD8-PfMATE complex was crystallized (Figure 2A) using the lipidic cubic phase (LCP) method [32]. The crystals were found to be in the space group C_2_, and no twinning defects were observed. Electron density was resolved to a 3.22 Å resolution, which turned out to be a modest improvement compared to the greater improvement in resolution obtained for the co-crystals with other macrocyclic peptides (MaL6 and MaD3S with the resolution with 2.45 Å and 2.6 Å, respectively) [29]. As a result, the atoms of the MaD8 peptide could not be accurately fitted into the electron density map, but the solved omit map and a rough fitting shown in Figure 2 clearly indicated MaD8’s binding site is located between the N- and C-lobes of PfMATE’s extracellular side. Although the resolution of the MaD8-PfMATE complex structure is lower than that of apo PfMATE, e.g., the P26A mutant is at 2.1 Å resolution, the subtle pH control over the conformational state of transmembrane helix 1, which allowed us to obtain the high resolution apo structures, was not known prior to the crystal structure elucidation of the conformationally-locked macrocyclic peptide-PfMATE complexes.

Unlike MaL6’s skewed cleft binding, which is possibly due to its voluminous ring size [29], MaD8’s smaller ring size allows for centered binding in the transporter’s cleft. Although MaL6 dominated the selections employing the ^L^F-library, the frequent appearance of clones bearing small rings in the later libraries of both libraries indicates that the peptides bearing smaller rings have an evolutionary advantage. This preference for smaller ring sizes appears to be quite unique when using PfMATE as a target. The selection against Akt2, which also used NNK-based macrocyclic peptide libraries, resulted in the isolation of peptides whose rings were composed of 10–14 amino acid residues despite the high potential for cysteine’s appearance in the random region, which would result in smaller ring sizes [25].

One of the main advantages of the use of Fv fragments [6] and larger Fab [7] CCPs is that new hydrophilic surfaces are not only introduced but also extended beyond the influence of the partial micelle. In the omit map of the MaD8-PfMATE complex, MaD8 is buried within the cleft between the two subdomains and does not appear to introduce hydrophilic surfaces or mediate any complex-to-complex contact. This suggests that the major contribution of the macrocyclic peptides with regards to facilitating co-crystallization is the stabilization of one conformation of PfMATE and not the enhancement of solubility or complex-to-complex contact.

### 2.3. Inhibition of Transporter Activity

As shown in Figure 2, MaD8 binds to the cleft of PfMATE’s extracellular side and helps lock the outward-open conformation. Since the transporter activity is dependent on conformational change, MaD8 appears to be a good candidate for an inhibitor of transport activity by preventing conversion to the inward-open state. In addition, by binding to the exit gate directly in the path of a substrate molecule, MaD8 may physically block substrate extrusion. To verify that MaD8 possesses inhibitory activity, we performed an accumulation assay using ethidium bromide (EtBr) as a translocation substrate.

**Figure 3:**
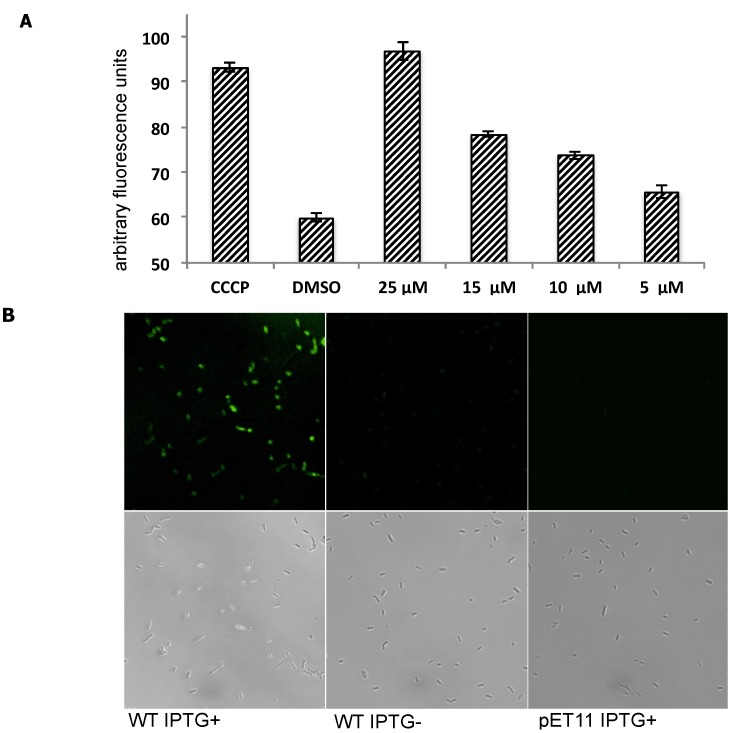
Inhibition of efflux activity of PfMATE using MaD8 and selective staining of PfMATE-expressing *E. coli* cells by fluorescein-labeled MaD8. (**A**) Inhibitory activity of MaD8. Graphs showing the increase in fluorescence after 10 min of incubation of *E. coli* cells, 50 µM EtBr and a chemical additive. The CCCP bar represents the total fluorescence of *E. coli* cells treated with 100 µM carbonyl cyanide m-chlorophenylhydrazone. The DMSO bar represents the total fluorescence of *E. coli* cells without CCCP or macrocyclic peptide treatment. The remaining bars represent the total fluorescence of *E. coli* cells treated with the indicated concentration of MaD8; (**B**) Selective staining. The respective IPTG-induced PfMATE-expressing *E. coli*, IPTG-uninduced *E. coli*, and PfMATE-deficient *E. coli* strains were stained with MaD8F at 30 µM and subsequently washed to remove unbound fluorescent peptide. The top panel images show the fluorescence images observed by using a laser confocal microscope, and the bottom panel images show live-cell images under a transmitted light.

EtBr was used as PfMATE’s substrate to indirectly monitor PfMATE’s efflux activity [33]. For this assay, EtBr was added to *E. coli* C41(DE3)∆acrB cells suspended in a pH-neutral buffer (pH 7.0). EtBr would enter the *E. coli* cells and intercalate into the genomic DNA, which causes an increase in the total fluorescence of the entire *E. coli* cell suspension. This change in fluorescence is proportional to the time-dependent change in the intracellular concentration of EtBr, and therefore, the time-dependent change in fluorescence of the suspension was measured as the EtBr concentration approaches a steady-state equilibrium. Since the intracellular concentration should be dependent on the rate of EtBr influx minus the rate of the EtBr efflux, a slow or no increase in fluorescence of the total *E. coli* cell suspension could be indicative of high PfMATE activity, which prevents EtBr from accumulating within the *E. coli* cells. Conversely, inhibition of PfMATE function by the presence of an inhibitor, in this case MaD8, should result in an elevated rate of EtBr accumulation. Indeed, pretreating PfMATE-expressing *E. coli* cells with MaD8 prior to the addition of EtBr resulted in an elevated rate of increase in fluorescence, suggesting that MaD8 is capable of binding to the extracellular side of membrane-bound PfMATE and inhibiting EtBr efflux in a MaD8 concentration-dependent manner (Figure 3A and Figure S4). MaD8’s inhibitory activity is, in fact, similar to that observed for MaL6, another cleft-binding peptide. At a concentration of 25 µM, both cleft-binding peptides promoted EtBr accumulation roughly equivalent to the EtBr accumulation promoted by 100 µM cyanide m-chlorophenylhydrazone (CCCP). However, both of these cleft-binding macrocyclic peptides are less potent than the substrate pocket-binding macrocyclic peptides such as MaD5 and MaD3S [29].

Because the PfMATE is specifically expressed in the inner-membrane of *E. coli* cells, the observed inhibitory activity against PfMATE implies that MaD8 penetrated the outer-membrane by an unknown mechanism and reached PfMATE in the inner-membrane. To verify this event indirectly, we synthesized a fluorescein-labeled derivative of MaD8 (MaD8F, Figure S5) and stained IPTG-induced PfMATE-expressing *E. coli* (Figure 3B, WT IPTG+), uninduced *E. coli* (WT IPTG−), and PfMATE-deficient *E. coli* (pET11 IPTG+) strains. MaD8F was able to stain PfMATE-expressing *E. coli* selectively, but did not significantly stain uninduced *E. coli* containing the PfMATE gene or PfMATE-deficient *E. coli* (Figure 3B).

## 3. Experimental

### 3.1. Preparation of *N*-ClAc-l-phenylalanine-tRNA^fMet^_CAU_ and *N*-ClAc-d-phenylalanine-tRNA^fMet^_CAU_

Initiator tRNA^fMet^_CAU_ (5.25 nmol) was charged with either *N*-(2-chloroacetyl)-l-phenylalanine or *N*-(2-chloroacetyl)-d-phenylalanine were charged onto according to literature protocols [20,24,25]. Charged tRNAs for the first rounds of the sub-selections were stored at −80 °C as dry pellets. For all other rounds, charged tRNAs were stored in 175 pmol aliquots at −80 °C as dry pellets.

### 3.2. Library Construction

The DNA library was constructed according to protocol with some modification [25]. mRNA libraries were transcribed from DNA constructed using oligonucleotides listed in Table S1. mRNA libraries that code for 7–15 random amino acids were used for the initial library. The mRNA libraries transcribed from the DNA libraries were mixed in the ratio 160:40:10:2.5:0.625:0.156:0.391:0.00977:0.00244 pmol for library templates NNK15:NNK14:NNK13:NNK12:NNK11:NNK10:NNK9:NNK8:NNK7, respectively, in a total volume of 100 µL.

### 3.3. Protein Immobilization

His-tagged PfMATE was prepared according to literature [29]. For the first rounds of the sub-selections, His-tagged PfMATE (751 pmol) was used to saturate his-tag binding sites on Invitrogen (Grand Island, NY, USA) Dynabeads^®^ His-Tag Isolation & Pulldown magnetic beads (600 µg) in HEPES-buffered saline (20 mM HEPES, 150 mM NaCl, 0.1% Cymal-6 (6-cyclohexyl-1-hexyl-β-D-maltose, w/v), pH = 7.0, 150 µL). For all other rounds, His-tagged PfMATE (50 pmol) was used to saturate his-tag binding sites on Invitrogen Dynabeads^®^ His-Tag Isolation & Pulldown magnetic beads (40 µg) in HEPES-buffered saline (20 mM HEPES, 150 mM NaCl, 0.1% Cymal-6 (w/v), pH = 7.0, 10 µL). The protein-bead mixtures were incubated with gentle rotation at room temperature for 20 min. After incubation, the beads were washed three times with HEPES-buffered saline (for Round 1, 300 µL and for Round 2^+^, 30 µL) to remove unbound PfMATE.

### 3.4. Production of the mRNA-Puromycin Conjugates for Round 1

For Round 1, a ligation reaction mixture (20% DMSO, 1× T4 RNA ligase buffer, 1.5 µM puromycin linker, 1 µM mRNA, 236 pmol of T4 ligase, 200 µL) was incubated at room temperature for 30 min. A stop solution (0.6 M NaCl, 10 mM EDTA, pH = 7.5, 200 µL) was added to halt the reaction. The solution was extracted with 25:24:1 phenol–chloroform–isoamyl alcohol (400 µL) followed by a second extraction with chloroform–isoamyl alcohol (400 µL). The mRNA-puromycin conjugates were precipitated with the addition of ethanol (800 µL) to the aqueous phase. The resultant precipitate was pelleted by centrifugation (13,000 rpm, 15 min), washed with 70% ethanol (400 µL) followed by centrifugation (13,000 rpm, 3 min) and air-dried. The precipitate was dissolved in water (30 µL).

### 3.5. Production of the mRNA-Puromycin Conjugates for Round 2^+^

For Round 2 and all subsequent rounds, a ligation reaction mixture (20% DMSO, 1× T4 RNA ligase buffer, 1.5 µM puromycin linker, 1 µM mRNA, 47.2 pmol of T4 ligase, 40 µL) was incubated at room temperature for 30 min. Water (40 µL) was added to the reaction. An 80 µL stop solution (0.6 M NaCl, 10 mM EDTA, pH = 7.5) was added to halt the reaction. The solution was extracted with 25:24:1 phenol–chloroform–isoamyl alcohol (160 µL) followed by a second extraction with 24:1 chloroform–isoamyl alcohol (160 µL). The mRNA-puromycin conjugate was precipitated with the addition of ethanol (320 µL) to the aqueous phase. The precipitate was washed with 70% ethanol and air-dried. The precipitate was dissolved in water (8 µL). 

### 3.6. Selection with the Binding Step at 4 °C

The first rounds of the *in vitro* sub-selections were performed using 200 pmol of mRNA-puromycin conjugate and 5.25 nmol of N-(2-chloroacetyl)-L-phenylalanine-tRNA^fMet^_CAU_ or N-(2-chloroacetyl)-D-phenylalanine-tRNA^fMet^_CAU_ in a FIT system with a reaction volume of 150 µL [14,23,24]. The translation reactions were incubated at 37 °C for 30 min followed by 12 min of incubation at room temperature. An EDTA solution (200 mM, 15 µL) was added to each translation reaction. The reactions were incubated again at 37 °C for 30 min to facilitate cyclization. Salts were removed by passing the translation reactions through Sephadex G-25 columns that were pre-washed with HEPES-buffered saline. 2× Blocking solution (1 M NaCl, 0.2% acetylated Bovine Serum Albumin, 20 mM HEPES, 150 mM NaCl, 0.1% Cymal-6 (w/v), pH = 7.0, 165 µL) was added to each of the desalted peptide-mRNA solutions. Undesired bead-binding peptide-mRNAs and his-tagged FIT system components were removed by incubating the peptide-mRNAs with storage buffer-free magnetic beads (6 mg). The supernatant was separated from the beads and 1 µL of supernatant was used for reverse transcription and real-time PCR to measure the concentration of the input peptide-mRNA. From the remaining supernatants (329 µL), 300 µL of the peptide-mRNA solution was incubated with PfMATE-beads (as prepared in the Protein Immobilization Section) for one hour at 4 °C with gentle rotation. After incubation, the supernatants were removed, and unbound peptide-mRNAs were removed from the magnetic beads by washing the beads three times with ice-cold HEPES-buffered saline (800 µL). To the beads, a reverse transcription reaction mixture (1.25× MMLV RT buffer, 0.625 mM dNTP, 3.125 µM primer CGS3an13.R39 (Table S1), 8 U RNasin ribonuclease inhibitor (Promega, Madison, WI, USA), 300 U M-MLV reverse transcriptase (Promega), 40 µL) was added. The reaction was incubated at 42 °C for 1 h. To the reverse transcription reactions including the beads, 200 µL of polymerase-free PCR Reaction MIX (1× Taq PCR Buffer, 2.5 mM MgCl_2_, 0.5 mM dNTP, 0.25 µM primer T7g10M.F48, 0.25 µM primer CGS3an13.R39 (Table S1)) were added and the mixture was incubated at 95 °C for 5 min. The supernatants were transferred into new tubes and 600 µL of polymerase-free PCR Reaction MIX and 6 µL of Taq DNA polymerase were added. For quantification of the peptide-mRNAs recovered by bead binding, 20 µL of this mixture was removed for use in real-time PCR. The remaining 826 µL of solution was thermocycled 16 × (95 °C/40 s, 50 °C/40 s, 72 °C/40 s). The PCR product was extracted with one equivalent volume of 25:24:1 phenol–chloroform–isoamyl alcohol followed by extraction using one equivalent volume of 24:1 chloroform–isoamyl alcohol. A solution of NaCl (3 M, 82 µL) was added to the aqueous phase and the DNA product was precipitated with the addition of ethanol (1,640 µL). The pellet was dissolved in KCl solution (50 mM, 30 µL). Dissolved DNA (4 µL) was added to 16 µL of a transcription reaction mixture (1.2× T7 buffer, 12 mM dithiothreitol, 24 mM MgCl_2_, 26.4 mM KOH, 4.5 mM NTPs, 4 U RNasin ribonuclease inhibitor (Promega), and 240 nM T7 polymerase). The transcription reaction was incubated at 37 °C overnight (>12 h).

All subsequent rounds were performed as above with modifications. *In vitro* translation reactions were performed using 175 pmol of *N*-(2-chloroacetyl)-l-phenylalanine-tRNA^fMet^_CAU_ or *N*-(2-chloroacetyl)-d-phenylalanine-tRNA^fMet^_CAU_ and 7.5 pmol of mRNA-puromycin conjugate in a total volume of 5 µL. To avoid the isolation of RNA aptamers with affinity towards the target protein, complementary DNA (cDNA) was reverse transcribed prior to the selection step. Selection incubation time was shortened to thirty minutes. One hundred microliters of polymerase-free PCR Reaction MIX was used during the melting of cDNA from the bound peptide-mRNA. The amount of bead-bound peptide-mRNAs was determined by taking a 1 µL aliquot from the supernatant containing single-stranded cDNA. The DNA molecules obtained after the 7th round of both libraries were ligated into the plasmid pGEM-T Easy using TA-cloning. Individual clones were picked arbitrarily. Sequencing was performed by FASMAC (Table 1).

### 3.7. Selection with the Binding Step at 37 °C

A second sub-selection was conducted according to the protocol for the sub-selection performed at 4 °C except that with the PfMATE-immobilization, pre-clearances, PfMATE binding, and all washings of the beads were performed using buffers and incubators equilibrated to 37 °C. The selection was halted after Round 6 (Figure S2C,D) and individual clones were sequenced (Table 1).

### 3.8. Selection Round Using a Chemically Synthesized Competitor

Using the library obtained after Round 5, MaL6-competitive rounds of this second selection at 37 °C (c-Round 6) were performed. The competitive condition included the addition of mRNA-free, chemically synthesized MaL6 (final concentration of 32 µM, *vide infra*) in the buffer of the binding step, and all other steps were performed identically to non-competitive Round 6. Individual clones were sequenced (Table 1).

### 3.9. Single-Clone Assays

The binding ability of individual clones was determined by using single-clone display (Figure S4). The assay conditions used were the same conditions as the second and subsequent rounds of each sub-selection.

### 3.10. Peptide Chemical Synthesis

Peptides were chemically synthesized with only a single glycine-carboxamide in place of the (Gly-Ser)_3_ linker used in the selection. MaL6, MaD8 and MaD8F were synthesized using an automated peptide synthesizer (Syro, Biotage, Uppsala, Sweden). For MaD8F, an additional beta-alanine and MMT-lysine were appended to the C-terminus to facilitate reaction with NHS-fluorescein (Thermo Scientific, Waltham, MA, USA). Peptides were cleaved from the resin and cyclized according to protocol [23]. Macrocyclic peptides were purified using HPLC (Imtakt Cadenza CD-C18 250 × 10 mm column, Gilson HPLC, Middleton, WI, USA). The mass was verified by MALDI-TOF mass spectrometry (Autoflex II, Bruker Daltronics, Fremont, CA, USA). MALDI-TOF: calcd. for C_114_H_148_N_23_O_25_S^+^ (MaL6): 2271.074; found 2271.009. MALDI-TOF: calcd. for C_83_H_125_N_26_O_22_S_2_^+^ (MaD8): 1901.890; found 1902.220. MALDI-TOF: calcd. for C_83_H_125_N_26_O_22_S_2_^+^ (MaD8F): 2459.070; found 2459.038.

### 3.11. Inhibition Assay

MaD8 was tested for inhibitory activity using ethidium bioaccumulation according to literature with modifications [33]. *E. coli* C41(DE3)∆AcrB was used to express His_6_-tagged PfMATE. Cells containing the PfMATE expression vector were grown in LB media containing 100 mg/L ampicillin overnight at 37 °C with shaking at 200 rpm. Stationary phase *E. coli* was diluted in a 100 times equivalent volume of fresh LB media containing 100 mg/L ampicillin. Cells were grown at 37 °C to an OD_600_ of 0.5, and expression was induced with the addition of IPTG (final concentration of 0.5 mM). Induced cells were incubated for 3 h at 37 °C with shaking at 200 rpm. Cells collected from 500 µL of induced culture were washed once with 50 mM Tris Buffer (500 µL, pH = 7.0) and resuspended in 50 mM Tris Buffer (pH = 7.0) to a final OD_600_ of 0.5. In the wells of a 96-well black plate (1/2 area, Perkin Elmer), 44.5 µL of the above cell suspension were added to 0.5 µL of peptide ranging in concentration from 0–2.5 mM in DMSO. For a positive control, 0.5 µL of a 10 mM solution of carbonyl cyanide m-chlorophenylhydrazone (CCCP) dissolved in DMSO was used instead of peptide dissolved in DMSO. Ethidium bromide (0.5 mM, 5 µL) was added to the *E. coli* cells mixed with peptide, a blank negative control, or a CCCP positive control, and the plate containing the mixtures was immediately placed into the plate reader (Flexstation 3, Molecular Devices, Sunnyvale, CA, USA). Excitation and emission wavelengths were 500 nm and 595 nm, respectively. Note that there is about a 15 s delay between addition of the ethidium bromide and the first reading (t = 0 s). Fluorescence readings were taken every 30-s interval from 0 to 1,800 s.

### 3.12. Staining *E. coli* Cells with MaD8F

IPTG-induced PfMATE-expressing *E. coli*, IPTG-noninduced *E. coli*, and PfMATE-deficient *E. coli* C41(DE3)∆AcrB cells were incubated with 30 µM MaD8F in a 10 µL volume of 50 mM Tris-HCl (pH = 7) at 37 °C and shaking at 200 rpm for 5 min. Cells were washed with 500 µL of 50 mM Tris-HCl (pH = 7) and visualized using a confocal microscope (TCS SP2, Leica, Concord, Canada).

### 3.13. Crystallization, Data Collection, and Structure Determination

Crystallization, data collection, and structure determination were performed according to literature with minor changes [26]. For the MaD8-PfMATE complex, the gel filtration buffer contained 20 mM NaCl, 20 mM HEPES pH = 7.0, 0.03% decyl-maltose neopentyl glycol and the crystallization reservoir solution contained 31% PEG400, 100 mM Li_2_SO_4_.

## 4. Conclusions

We demonstrated that the thioether-macrocyclized peptide, MaD8, selected by the RaPID system is capable of being cocrystallized with PfMATE and inhibits transport function, which suggests conformation specific binding of the outward-open state in solution phase. The robustness of the RaPID system for selection conditions allowed us to generate various macrocyclic peptides that bound to different regions of PfMATE, increasing the chances of identifying macrocyclic peptides capable of effectively locking flexible regions. Moreover, various shapes of macrocycles fit to a certain cavity between the transmembrane helices and thus inhibit the drug transporter function of PfMATE. The selection strategy of macrocyclic peptides reported herein is not limited to this particular transmembrane protein, but can be readily extended to various proteins which have been difficult to obtain crystals with satisfactory resolutions and their inhibitors.

## Supplementary Materials

Supplementary materials can be accessed at: http://www.mdpi.com/1420-3049/18/9/10514/s1.
