# Supplementary material for: A Macrocyclic Peptide that Serves as a Cocrystallization Ligand and Inhibits the Function of a MATE Family Transporter
Source: Molecules. 2013 Aug 30;18(9):10514–30. doi: 10.3390/molecules180910514 (PMC6270235; doi:10.3390/molecules180910514)

Supplementary Materials

Table S1. Oligonucleotides used in the selection.

Oligonucleotides used for selection	Sequence (5' -3')
T7g10M.F48	TAATACGACTCACTATAGGGTTAA*CTTTAAGAAGGAGATATACATATG*
NNK_n	**GCTGCCGCTGCCGCTGCCGCA**$(MNN)_n$*CATATGTATATCTCCTTCTTAAAG*
CGS3an13.R39	TTTCCGCCCCCCGTCCTA**GCTGCCGCTGCCGCTGCCGCA**
puromycin linker	d(pCTCCCGCCCCCCGTCC)-(SPC18)5-d(CC)-puromycin

Table S2. X-ray data collection.

**Data Collection**	PfMATE + D8	
Wavelength (Å)	0.91000	
Space group	*H*3	
Unit cell parameters (Å, °)	*a* = 152.8, *b* = 152.8<br>*c* = 163.0	
	α = 90.0, β = 90.0<br>γ = 120.0	
Resolution (Å)	50-3.22 (3.36-3.22)	
Unique reflections	23064	
Total reflections	869988	
Completeness	100 (100)	
$I/\sigma(I)$	4.34 (1.95)	
Redundancy	12 (9.0)	
R_{sym}	0.390 (0.648)	
**Refinement**		
Resolution (Å)	47.8-3.22	
No. reflections	22937	
R_{work}/R_{free}	0.280/0.317	
	(0.391/0.393)	
No. atoms	5857	
protein	5799	
cyclic peptide	58	
water	7	
B-factors		
protein	73.6	
cyclic peptide	226	
water	11.5	
R.m.s deviations		
Bond length (Å)	0.003	
Bond angles (°)	0.833	

The numbers in parentheses are for the highest resolution shell. *$R_{sym} = \Sigma|I_{avg}-I_i| / \Sigma I_i$. †$R_{cullis} = \Sigma|E|/\Sigma||F_{PH}|-|F_P||$, where F_{PH} is the amplitude of the protein plus the heavy atom and F_P is the amplitude of the protein. ‡Phasing power = r.m.s. ($|F_H|/E$), where $|F_H|$ is the heavy atom structure-factor amplitude and E is the residual lack of closure error. ¶Figure of merit = $\langle|\Sigma P(\alpha)\exp(i\alpha)/\Sigma P(\alpha)|\rangle$, where α is the phase and $P(\alpha)$ is the phase probability distribution.

Figure S1. Genetic code reprogramming and the FIT system. (**A**) The reprogrammed genetic code used for the initiation of ribosomal peptide synthesis; (**B**) Charging of initiator tRNA$^{fMet}_{CAU}$ with either *N*-(2-chloroacetyl)-L-phenylalanine or *N*-(2-chloroacetyl)-D-phenylalanine using flexizyme (eFx). Cyclization occurs spontaneously upon incorporation of a downstream cysteine; (**C**) Schematic representation of one round of *in vitro* selection from round 2 or a higher round.

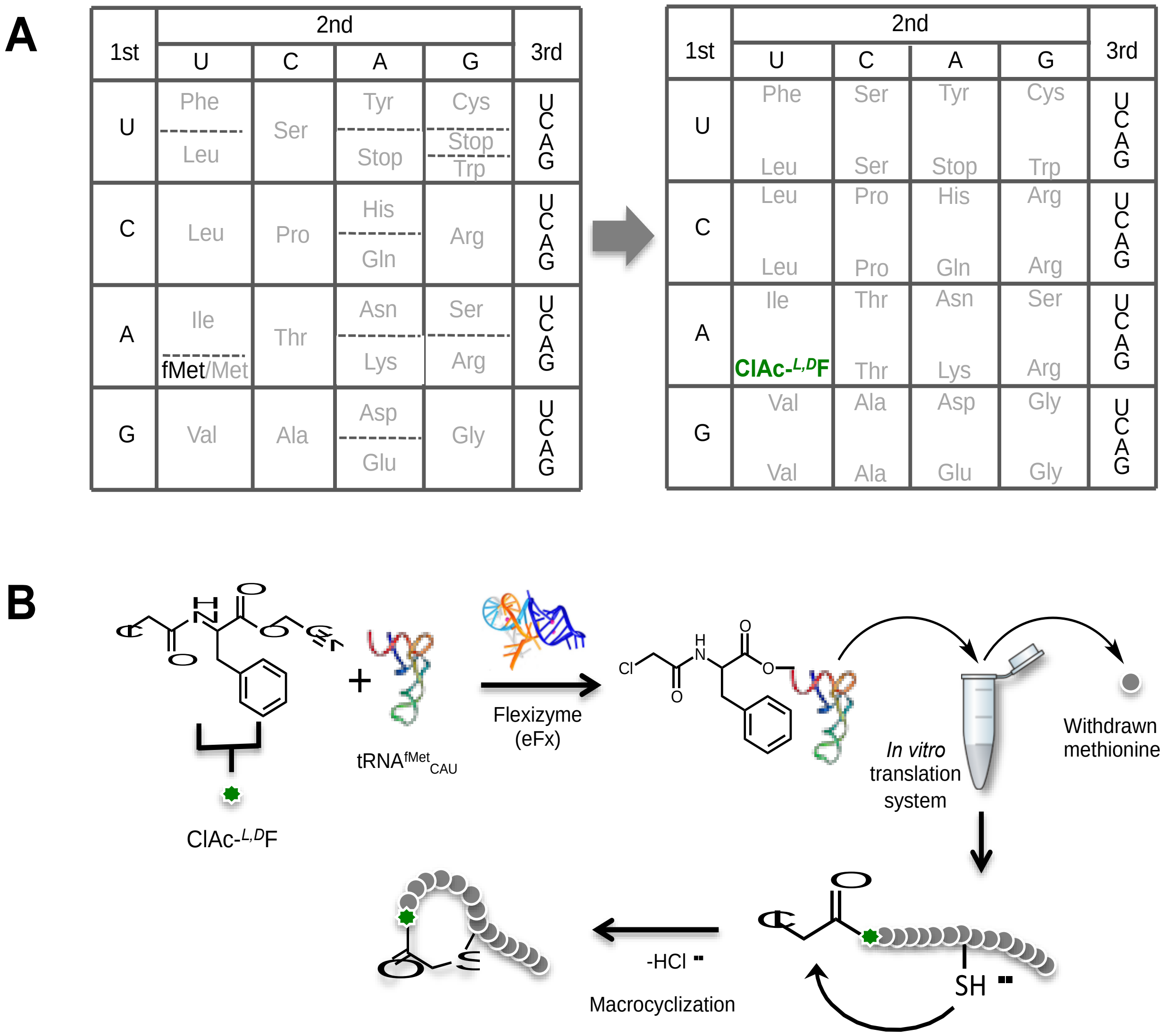

Figure S1. *Cont.*

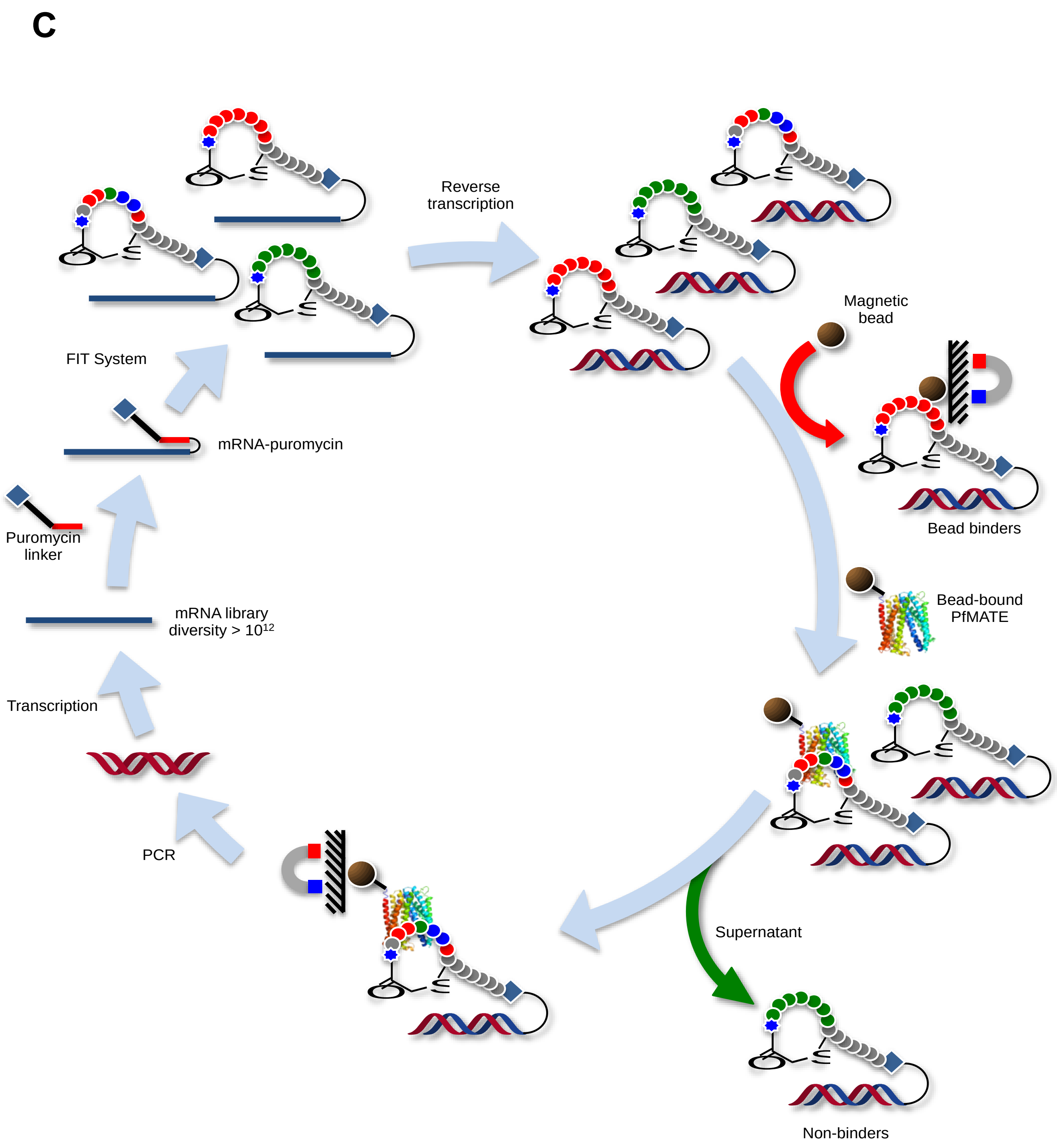

Figure S2. Progress of the selections. The progress of the selections whose binding step was performed at 4 °C of the (**A**) ^{L}F-Library and (**B**) ^{D}F-Library. The progress of the selections whose binding step was performed at 37 °C of the (**C**) ^{L}F-Library and (**D**) ^{D}F-Library. The rounds represented farthest to the right are the competition rounds, c-Round 6. Percentages of peptides bound were determined by dividing the amount of recovered cDNA by the amount of input macrocyclic peptide-mRNA conjugate.

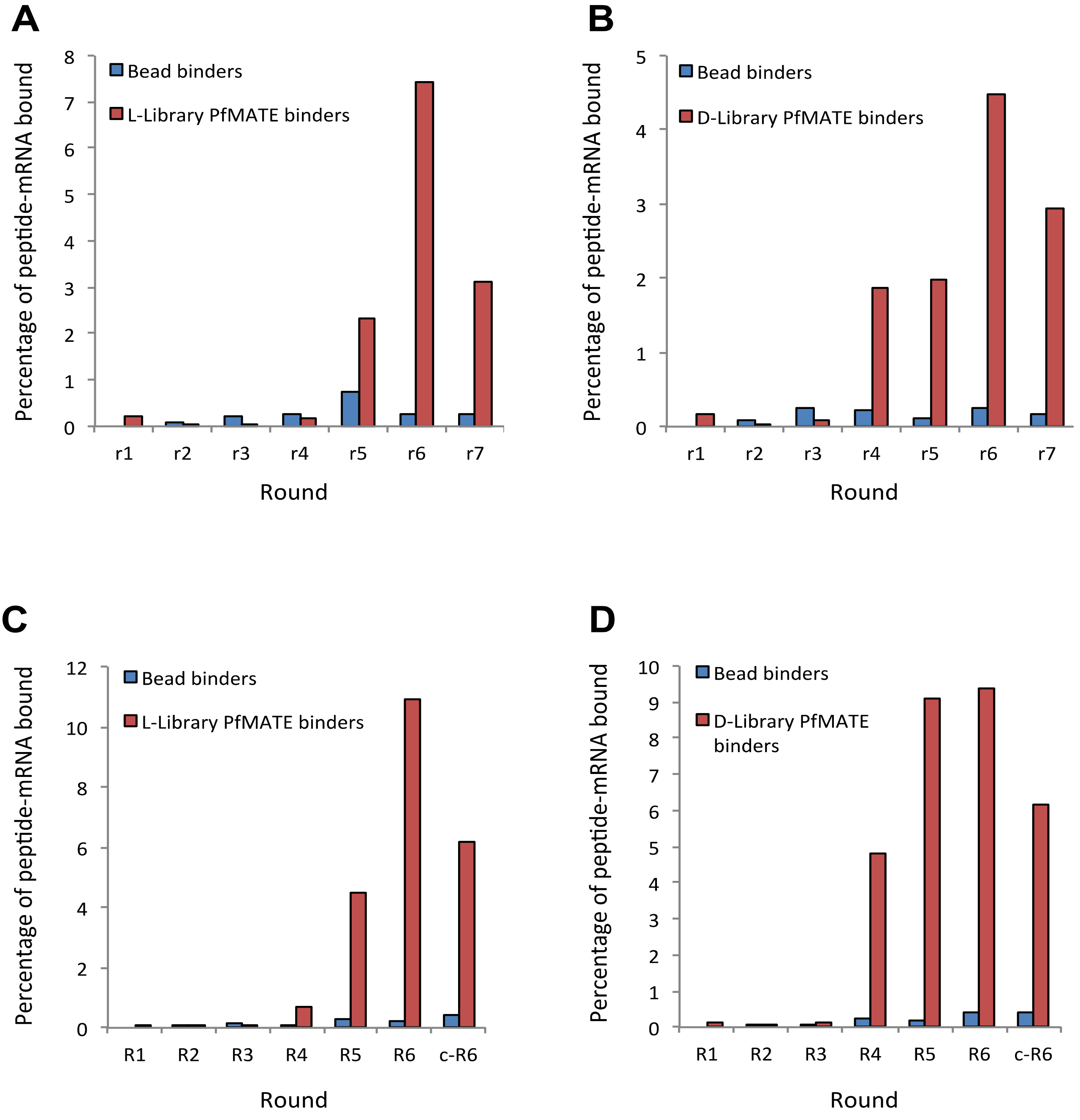

Figure S3. Percentages of peptides bound from single-clone display assays.

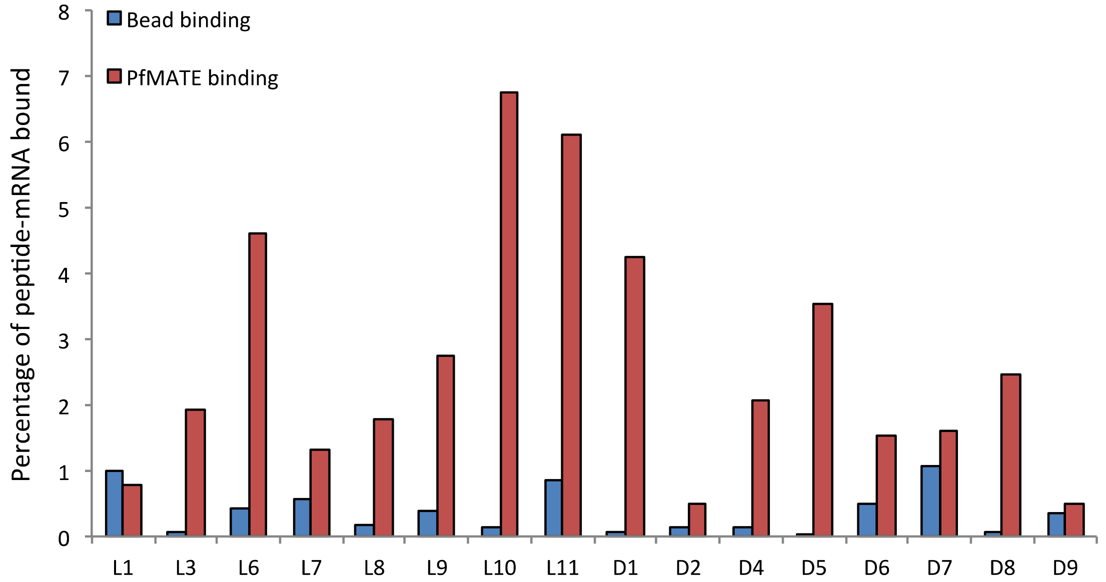

Figure S4. MaD8 concentration-dependent increase in the rate of accumulation of intracellular EtBr. Errors bars were calculated from four separate trials.

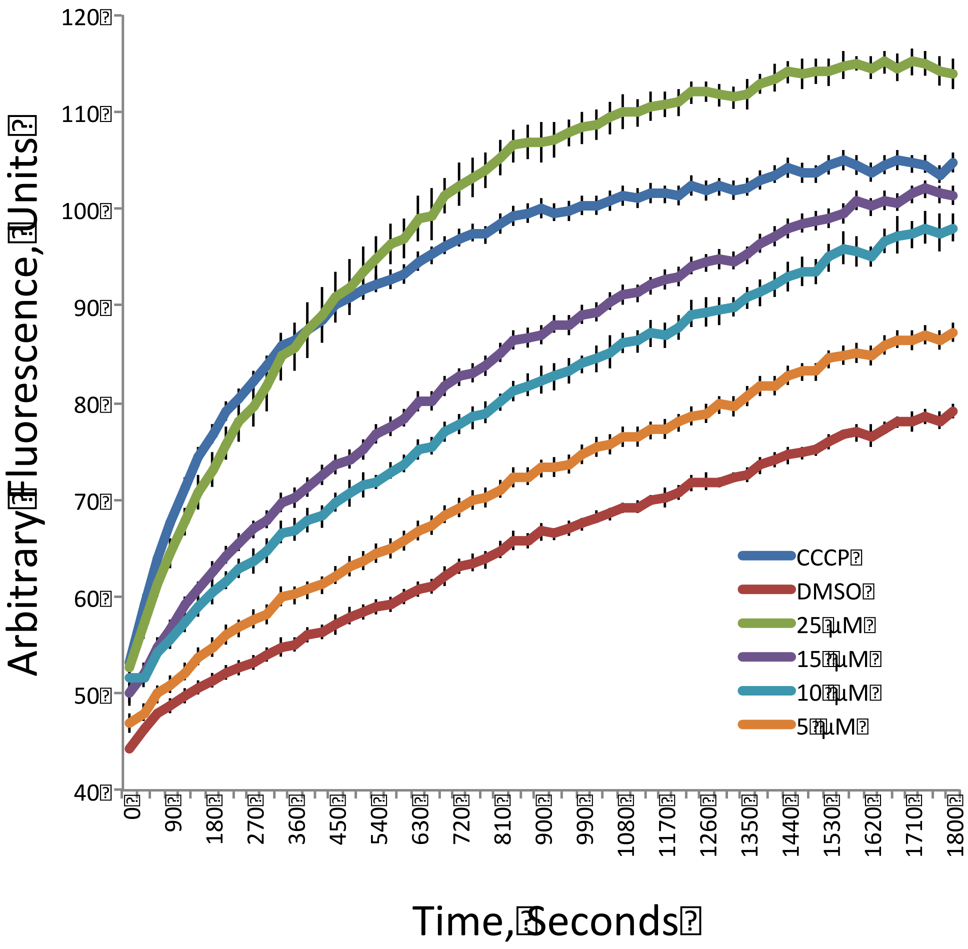

Figure S5. Chemical structure of MaD8F.

MaD8F

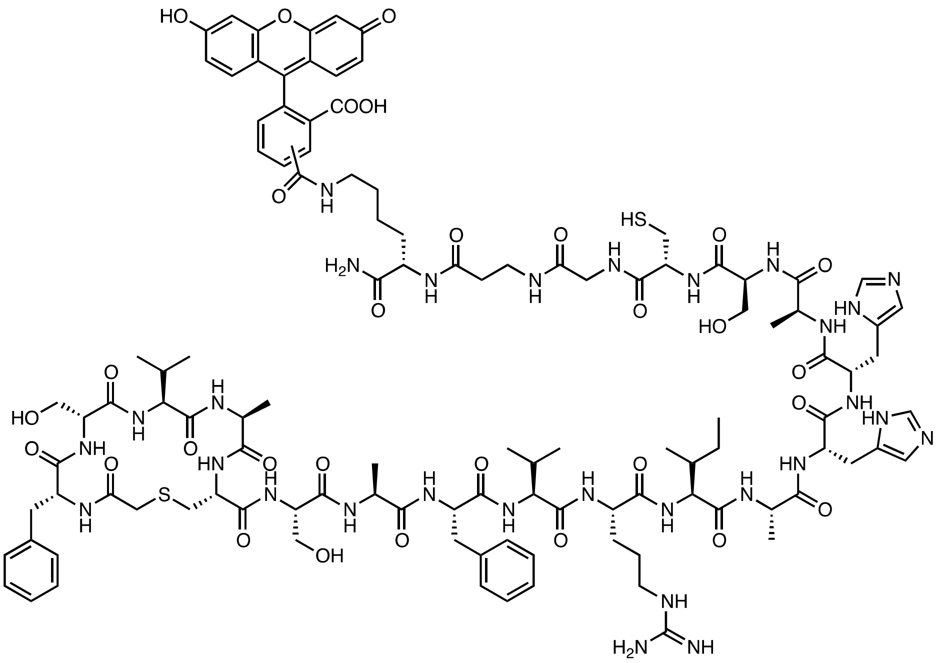

Supplement: Supplementary file 1 [file molecules-18-10514-s001.pdf]
